# Efficient Induction of Wheat-*Agropyron cristatum* 6**P** Translocation Lines and GISH Detection

**DOI:** 10.1371/journal.pone.0069501

**Published:** 2013-07-02

**Authors:** Liqiang Song, Lili Jiang, Haiming Han, Ainong Gao, Xinming Yang, Lihui Li, Weihua Liu

**Affiliations:** National Key Facility for Crop Gene Resources and Genetic Improvement/Institute of Crop Sciences, Chinese Academy of Agricultural Sciences, Beijing, China; Ben-Gurion University, Israel

## Abstract

The narrow genetic background restricts wheat yield and quality improvement. The wild relatives of wheat are the huge gene pools for wheat improvement and can broaden its genetic basis. Production of wheat-alien translocation lines can transfer alien genes to wheat. So it is important to develop an efficient method to induce wheat-alien chromosome translocation. *Agropyron* *cristatum* (**P** genome) carries many potential genes beneficial to disease resistance, stress tolerance and high yield. Chromosome 6**P** possesses the desirable genes exhibiting good agronomic traits, such as high grain number per spike, powdery mildew resistance and stress tolerance. In this study, the wheat- *A* *. cristatum* disomic addition was used as bridge material to produce wheat- *A* *. cristatum* translocation lines induced by ^60^Co-γirradiation. The results of genomic *in situ* hybridization showed that 216 plants contained alien chromosome translocation among 571 self-pollinated progenies. The frequency of translocation was 37.83%, much higher than previous reports. Moreover, various alien translocation types were identified. The analysis of M_2_ showed that 62.5% of intergeneric translocation lines grew normally without losing the translocated chromosomes. The paper reported a high efficient technical method for inducing alien translocation between wheat and *Agropyron* *cristatum* . Additionally, these translocation lines will be valuable for not only basic research on genetic balance, interaction and expression of different chromosome segments of wheat and alien species, but also wheat breeding programs to utilize superior agronomic traits and good compensation effect from alien chromosomes.

## Introduction

As the largest cereal crop worldwide, common wheat (*Triticum aestivum* L., 2*n* =6*X* = 42) plays an important role in the food security [1]. In recent years, modern cultivation systems have given rise to the sharp loses of wheat genetic diversity. Genetic erosion makes wheat increasingly vulnerable to biotic and abiotic stresses [2–5]. On the other hand, the increase of world population and the decrease in acreage of farmland require a much higher wheat yield to meet the need of humankind. The narrow genetic background has been the major barrier to wheat breeding. Importing alien excellent genes is the main method to widen the genetic basis of wheat. The wild relatives of wheat have an abundance of genetic diversity and desirable traits that are deficient in cultivated wheat [6,7]. *Lophopyrum* *ponticum* chromosome 7E possesses the leaf rust resistance gene *Lr19* and Fusarium head blight (FHB) resistance quantitative trait loci (QTL) [8]; *Psathyrostachys* *huashanica* chromosome 3Ns carries the gene(s) for resistance to stripe rust [9]. Powdery mildew resistance gene *Pm21* is located on the chromosome 6VS of *Haynaldia* *villosa* [10]. The gene(s) of high numbers of florets and kernels per spike is(are) located on chromosome 6**P** of *Agropyron* *cristatum* [11]. Introducing these genes to wheat has theoretical and practical significance in wheat germ plasm enrichment and cultivar improvement.

Intergeneric translocation can import alien chromosome segments or useful genes of the wild relatives into recipient wheat [12]. The wheat-rye 1**B**/1**R** translocation line is one of the most successful examples in the utilization of alien chromosome translocation: it not only improves wheat disease resistance, but also increases wheat production [13]. Strategies for producing wheat-alien translocations consist of *Ph*-mutation, tissue culture, *Aegilops* ’ gametocidal chromosomes and irradiation. The frequency of translocation induced by *Ph* gene mutants or tissue culture was usually less than 1% [14], and that induced by gametocidal chromosomes or irradiation was generally no more than 10% [7,14–16]. Therefore, it is very desirable to develop a more efficient method to induce wheat-alien chromosomal translocation for speeding up the gene exchanges between wheat and its related species.

*Agropyron* Gaertn. (**P** genome), one genus of wild relatives of wheat, has many superior traits including resistance to diseases [17,18]. Li et al [19–21] synthesized a series of intergeneric hybrids through wide hybridization and embryo rescue, and then obtained an array of wheat- *A* *. cristatum* addition lines. They had notable morphological differences, such as plant height, grain number per spike and the seed size, and showed resistance to powdery mildew and other diseases [21]. Compared with the recipient parent ‘Fukuhokomugi’, the addition line 4844-12 has the characteristics of superior numbers of florets and kernels per spike. Wu et al [11] located the gene(s) on the chromosome 6**P** of *A* *. cristatum* . Many reports paid more attention to the transfer of genes for disease resistance than the introgression of high-yield traits. Luan et al [7] acquired 23 wheat- *A* *. cristatum* 6**P** translocation plants induced by irradiation or *Aegilops* ’ gametocidal chromosomes. However, the translocation frequency was low and the number of complementary translocation was relatively small.

In this study, the wheat- *A* *. cristatum* disomic addition plants were irradiated by ^60^Co-γray and their self-pollinated progenies were characterized by genomic in situ hybridization (GISH). The objectives were (1) to develop an efficient method for inducing chromosomal translocations, which can provide a reference for the high-frequency induction between wheat and its related species; (2) to obtain various types of alien translocation, which can provide the basic materials for efficient utilization in wheat breeding and the research on genetic expression of alien chromosome fragment(s)/gene clusters in the wheat background.

## Materials and Methods

### Experimental materials

Wheat- *A* *. cristatum* 6**P** disomic addition line 4844-12(2*n* = 44), obtained by hybridization between *A* *. cristatum* accession Z559 (2*n* = 4*X* = 28, **PPPP**) from Xinjiang, China, and *Triticum aestivum* cv. ‘Fukuhokomugi’(2*n* = 6*X* = 42, **AABBDD**), is inherited stably and has obvious characteristics of multikernel (high numbers of florets and kernels per spike) [11].

### Induction techniques

Wheat- *A* *. cristatum* addition line were overwintered in the field and transplanted into pots before jointing. The plants at the stages of booting, heading and flowering were irradiated with^60^ Co gamma rays at a dose of 20 Gray (Gy) and a dose rate of 0.5 Gy/min at the cobalt source chamber of Peking University. Two repeats were performed with 75 plants per repeat. The seeds were harvested from irradiated plants. The non-irradiated addition line was used as control.

### Chromosome preparation

M_1_ Plant root tips were preserved at 70% ethanol after the treatment of low temperature and Carnoy. The chromosome slides were dealt with 45% glacial acetic acid [22]. Cytological observations were operated under a BX51 Olympus phase-contrast microscope (Olympus Corp., Tokyo, Japan) and the images were taken with a digital camera. The slides were frozen in liquid nitrogen at least 10 minutes and stored at -20°C until needed for GISH detection.

### GISH detection

Genomic in situ hybridization (GISH) was carried out in root tip cells to identify the translocations of M_1_ plants. The Biotin-Nick Translation Mix, Digoxigenin-Nick Translation Mix, avidin-fluorescein and anti-digoxigenin-rhodamine were purchase from Roche. The P-genomic DNA and ‘Fukuhokomugi’ genomic DNA were used as probe and block, at 1:40 ratio, respectively, to identify the *A* *. cristatum* chromosomal fragments. The GISH procedure followed that described by Liu et al. [15]. The GISH images were observed under a Nikon Eclipse E600 (Japan) fluorescence microscope and captured with a CCD camera (Diagnostic Instruments, Inc., Sterling Heights, MI, USA).

## Results

### The GISH detection of the M_1_ irradiated progenies of Wheat-*A*. *cristatum* 6P disomic addition line 4844-12

The genomic in situ hybridization (GISH) was used to identify 571 M_1_ irradiated progenies, of which 216 plants exhibited the wheat- *A* *. cristatum* chromosomal translocation, resulting in a translocation frequency of 37.83% (Table 1). At the same time, no chromosome structural change was detected in 30 non-irradiated plants (control), each one of them had two intact 6**P** chromosomes. This indicated that the chromosome translocation resulted from irradiation effect.

**Table 1:** GISH detection of M_1_ progeny.

Treatments	No. of plants observed	No. of translocation lines	Frequency of translocation (%)
First repeat	261	104	39.85
Second repeat	310	112	36.13
In total	571	216	37.83

### The alien translocation types and frequencies of the M_1_ progeny

The translocation types and frequencies between wheat and *A* *. cristatum* chromosomes are shown in table 2. The translocation plants were divided into two categories: the translocation plants with or without an intact chromosome 6**P**.

**Table 2:** The translocation types and frequency of wheat-*A. cristatum* chromosome 6**P** translocation.

Type of translocation line		First repeat	Second repeat	In total
		No. of translocation lines	No. of translocation lines	
Contain intact chromosome 6P	Whole-arm	14 (5.36%)	8 (2.58%)	22 (3.85%)
	Small fragmental	27 (10.34%)	22 (7.10%)	49 (8.58%)
	Large fragmental	12 (4.60%)	9 (2.90%)	21 (3.68%)
	Large and small fragmental	18 (6.90%)	21 (6.77%)	39 (6.83%)
	intercalary	1 (0.38%)	0	1 (0.18%)
	small fragmental and intercalary	3 (1.15%)	1 (0.32%)	4 (0.70%)
	small fragmental and whole-arm	2 (0.77%)	4 (1.29%)	6 (1.05%)
Without intact chromosome 6P	Whole-arm	2 (0.77%)	3 (0.97%)	5 (0.88%)
	Large fragmental	2 (0.77%)	4 (1.29%)	6 (1.05%)
	Small fragmental	10 (3.83%)	12 (3.87%)	22 (3.85%)
	intercalary	1 (0.38%)	0	1 (0.18%)
	Large and small fragmental	9 (3.45%)	24 (7.74%)	33 (5.78%)
	small fragmental and whole-arm	0	1 (0.18%)	1 (0.18%)
	small fragmental and intercalary	0	3 (0.53%)	3 (0.53%)
	Large and small fragmental, intercalary	2 (0.77%)	0	2 (0.35%)
	Large and small fragmental, whole-arm	1 (0.38%)	0	1 (0.18%)
In total		104 (39.85%)	112 (36.13%)	216 (37.83%)

Note: large fragmental translocation (W–P.P): chromosome 6**P** segment is more than one arm, the chromosome contains the centromere of 6**P**; small fragmental translocation (W–W.P): chromosome 6**P** segment is less than one arm, the chromosome contains the wheat centromere; whole-arm translocation: both the arms of translocated chromosome are from wheat and *A* *. cristatum* respectively; intercalary translocation: chromosome 6**P** segment is inserted into wheat chromosome arms.

**Figure 1:**
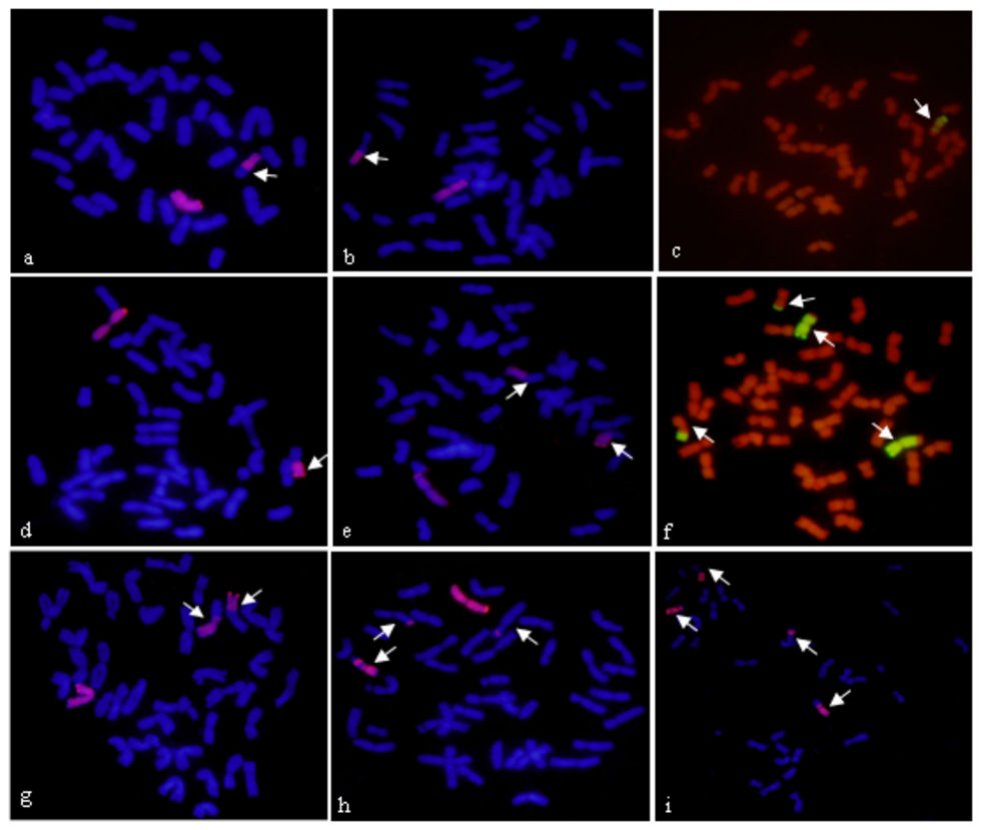
GISH detection of root tips of M_1_ Plant. a, b, c, e, g, h, i: The P-genomic DNA signal is red, while wheat DNA is stained blue by DAPI. c, f: The P-genomic DNA signal is green, while wheat DNA is stained red by PI. Arrows point to the different types of alien translocated chromosomes **a** Large fragmental translocation and a 6**P**; **b** Small fragmental translocation and a 6**P**; **c** Large fragmental translocation; **d** Whole-arm translocation and a 6**P**; **e** Whole-arm reciprocal translocation and a 6**P**; **f** Large fragmental and small fragmental reciprocal translocation; **g** Large fragmental, small fragmental translocation and a 6**P**; **h** Small fragmental intercalary translocation and a 6**P**; **i** Large fragmental, small fragmental and intercalary translocation

The alien translocation plants with one intact 6**P** chromosome were 142 (24.87%), consisting of 22 plants (3.85%) with a whole-arm translocation, 21 plants (3.68%) with a large fragmental translocation containing the 6**P** centromere (Figure 1a), 49 plants (8.58%) having a small fragmental translocation containing the wheat centromere (Figure 1b), 39 plants (6.83%) with both a large and a small fragmental translocation (Figure 1g). These translocation lines had higher translocation frequency. In addition, 1 plant (0.18%) had intercalary translocation, 4 plants (0.70%) had both small fragmental and intercalary translocations (Figure 1h), 6 plants (1.05%) had a small fragmental and a whole-arm translocation. These translocation lines had lower translocation frequency. Among 22 whole-arm translocation plants, 14 plants had either long-arm or short-arm of 6**P** translocated into wheat (Figure 1d), and 8 plants had both long-arm and short-arm of 6**P** translocated into wheat (Figure 1e).

There were 74 (12.96%) alien translocation plants without the intact 6**P** chromosome, including 5 plants (0.88%) with a whole-arm translocation, 6 plants (1.05%) with large fragmental translocation (Figure 1c), 22 plants (3.85%) having small fragmental translocation, 1 plant (0.18%) having intercalary translocation, 33 plants (5.78%) having both large and small fragmental reciprocal translocations (Figure 1f), 1 plant (0.18%) having small fragmental and whole-arm double translocations, and 3 plants (0.53%) with both small fragmental and intercalary double translocations. In addition, 3 plants had complex translocation, including 2 plants (0.77%) with large fragmental, small fragmental and intercalary triple translocations (Figure 1i), and 1 plant (0.18%) with large fragmental, small fragmental and whole-arm triple translocations.

**Figure 2:**
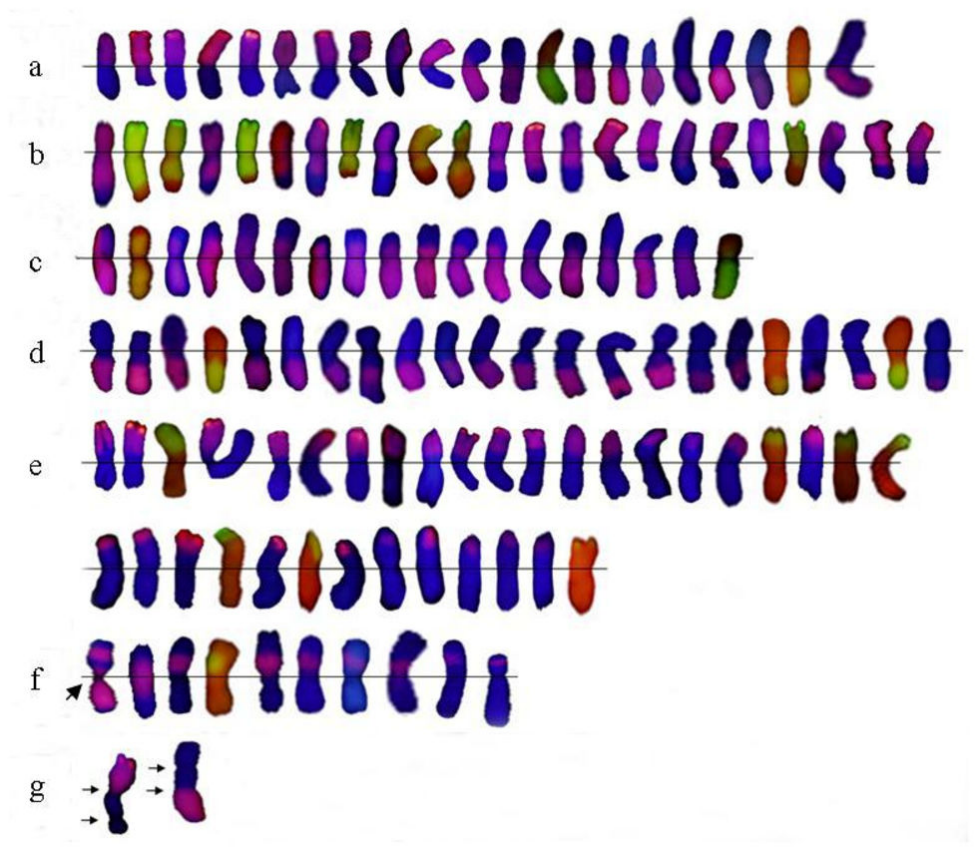
The translocated chromosomes of wheat-*A. cristatum* chromosome 6P. a: The **P**-genomic DNA signal is red (wheat DNA is blue stained by DAPI), b: The **P**-genomic DNA signal is green(wheat DNA is red stained by PI). a whole-arm translocation, b～c large fragmental translocation, d～e small fragmental translocation, f intercalary translocation, g dicentric translocation

The alien translocated chromosomes detected by GISH were shown in Figure 2. Figure 2a showed whole-arm translocated chromosomes, and their breakage points were at the centrosome. Figure 2b exhibited large fragmental translocated chromosomes with 6**P** centrosome, and their breakage points were at the long arm of chromosome 6**P** (W-6PL.S). Figure 2c displayed large fragmental translocated chromosomes with 6**P** centrosome, and their breakage points were at the short arm of chromosome 6**P** (W-6PS. L). Figure 2d showed small fragmental translocated chromosomes with wheat centrosome, and their breakage points were at the long arm of chromosome 6P (W. W-6PL). Figure 2e exhibited small fragmental translocate chromosomes with wheat centrosome, and their breakage points were at the short arm of chromosome 6**P** (W.W-6PS). Figure 2f displayed intercalary translocated chromosomes. The chromosome pointed by arrow had complex recombination with two fragments (red color), formed by four fractures, of chromosome 6**P** fused with three wheat fragments (blue color). Figure 2g showed a pair of dicentric translocated chromosomes.

The alien segments of translocated chromosomes differed in length. It revealed that irradiation treatment caused chromosome breakages randomly and the breakpoints also tended to distribute at random. The frequency of one break and fusion event was far higher than two or more events. Moreover, the break in interstitial regions is more frequent than that in the centric regions.

### Alien translocation produced by irradiating spikes at different developmental stages

According to the development stage, the spikes to be irradiated were divided into three types: booting, heading and flowering. Effect of irradiation treatment at different stages varied considerably (Table 3). The frequency of alien translocation at the stages of booting, heading and flowering were 28.34%, 44.07% and 61.25%, respectively. Thus, the frequency of alien translocation at flowering stage is the highest, suggesting that the gametes in the flowering stage are most sensitive to irradiation. Analysis of variance and Duncan’s multiple range test using the SAS system showed that the translocation frequencies of irradiated spikes at varying developmental stages were significantly different (P=0.0027, α = 0.05).

**Table 3:** Frequency of alien translocation of irradiated spikes at three stages.

Developmental stage of spike		First repeat			Second repeat			In total	
		No. of plants	No. of translocation plants		No. of plants	No. of translocation plants		No. of plants	No. of translocation plants
Booting (c)		142	42 (29.58)		172	47 (27.33)		314	89 (28.34)
Heading (b)		83	39 (46.99)		94	39 (41.49)		177	78 (44.07)
Flowering (a)		36	23 (63.89)		44	26 (59.09)		80	49 (61.25)
In total		261	104 (39.85)		310	112 (36.13)		571	216 (37.83)

Note: booting: young panicle hasn’t been exposed from leaf sheath; heading: the spike has been exposed from leaf sheath but not flowering yet; flowering: the pollen has been shed from anthers.

The values followed by a b or c within the same column are significantly different at P=0.005

### Transmission analysis of translocated chromosomes from M_1_ to M_2_ generation

**Figure 3:**
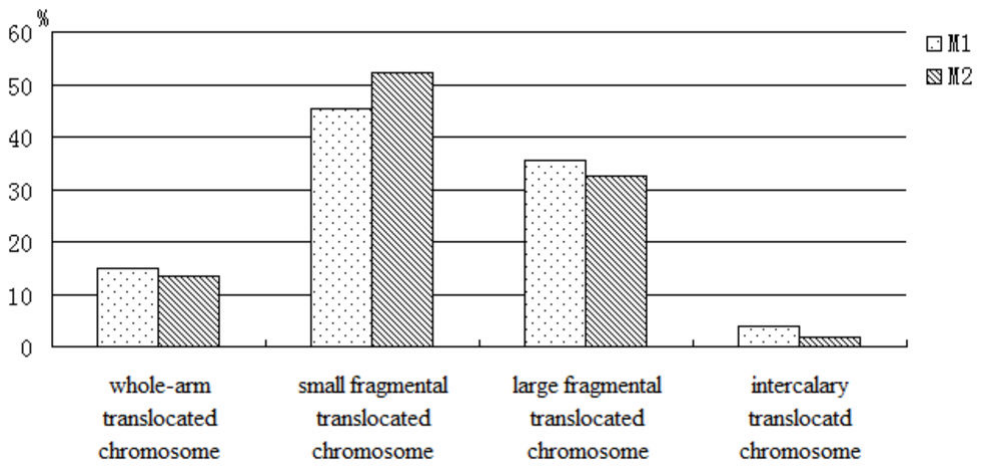
The ratio of different types of translocated chromosome in M_1_ and M_2_ generation.

Among the translocated lines detected in M_1_ generation, 62.5% plants grew normally and produced seed by self-pollinating. The ratio of the types of translocated chromosome in M_1_ and M_2_ generation was calculated (Figure 3). The number of small fragmental translocated chromosome was highest accounting for 45.45% of all translocated chromosome in M_1_. The ratio of small fragmental translocated chromosome increased to 52.38% in M_2_. However, the ratios of whole-arm translocated chromosome, large fragmental translocated chromosome and intercalary translocated chromosome reduced slightly in M_2_ generation. These were 15.04%, 35.66%, 3.85% respectively in M_1_, and 13.42%, 32.47%, 1.73% respectively in M_2_. This indicated that small fragmental translocatd chromosome had higher transmission ability than other types of translocated chromosome.

## Discussion

### Significance of the highly efficient induction of wheat-alien translocation

In previous reports, the frequency of wheat- *A* *. cristatum* translocation induced by gametocidal chromosome was 3.75% [15], and 5.08%, 2.78%, 2.12% by means of gametocidal chromosome, irradiated hybrids and irradiated pollen, respectively [7]. In this study, when the wheat- *A* *. cristatum* 6**P** disomic addition plants were irradiated by ^60^Co-γray at the flowering stage, the frequency of alien translocation was 37.83%, the hightest ever reported. We treated other wheat- *A* *. cristatum* lines by this method and got similar translocation frequency (unpublished results). In this study, most of the translocated plants could grow normally and produce seed by self-pollinating. A small part of translocated plants were inferior in the self-pollinated seed and transmission of translocated chromosome. This situation might be a result of (1) a large segment of wheat chromosome was replaced by a 6**P** chromosomal segment that did not have good compensation or (2) the plant was somewhat injured by irradiation affecting the function of the gametes (especially the pollen). Backcrossing of M_1_ plants with normal pollens of common wheat contributed to the normal seed set of the translocated plants and the normal transmission of the translocated chromosomes.

Dry seed, plant at meiosis and spike at pollen stage can be used as irradiated materials [23], while the frequency of translocation through irradiated plants at meiosis or spikes at pollen stage is much higher than that of irradiated dry seeds [24]. Gametes are more sensitive to irradiation and easier to generate chromosomal changes. Due to the protection of ovary, female gamete was usually more recalcitrant than male gamete to irradiation treatment. Seventy four alien translocated plants (34.26% of all translocated plants) did not have an intact chromosome 6**P** in our study. It was inferred that both male and female gametes generated a certain degree of variation and chromosomal translocation, which might be a reason why we got the high translocation frequency.

The strategies for inducing alien translocation include *Ph* gene manipulation, gametocidal chromosomes and irradiation. The *Ph* gene manipulation results in a low frequency of induced translocation and could only use the ‘Chinese Spring’ wheat; the gametocidal chromosomes also exist in the ‘Chinese Spring’ background, which gives inferior agronomic traits to the progeny. Since Sears [25] used irradiation treatment to transfer a gene for resistance to leaf rust to wheat chromosome 6B, the method has been widely adopted to induce chromosome translocation. Irradiation has several advantages: the breakage occurs at random, many translocation types could be obtained; theoretically, any alien segment could be inserted into wheat chromosomes without losing any wheat chromatin [2]. Therefore, the method of irradiating plants at anthesis was selected to generate chromosomal translocation.

Producing wheat-alien translocation is the best way of using desirable gene(s) in wild relatives to improve wheat. The improvement of the alien translocation induction frequency is significant for speeding up the exchange of genes between species, screening innovative germplasms from various translocation types and other theoretical research (e.g., positioning exogenous gene). The mass production of intergeneric translocation can provide basic materials for developing specific molecular and physical map construction of *A* *. cristatum* chromosome 6**P**. In addition, these translocation lines lay the foundation for the fine mapping and cloning of useful genes carried by chromosome 6**P**. Moreover, small fragmental translocation plants, especially intercalary translocation, make better use of alien gene(s) [26]. In this study, 160 plants (28.28%) contained small fragmental translocation and 11 plants (1.93%) contained intercalary translocation. Compared to large fragmental translocation, these small translocated chromosomes could be easily transmitted to the progeny.

### Genetic implication of various types of alien translocation obtained

Luan et al [7] acquired 23 wheat- *A* *. cristatum* translocation plants, nearly half of them were whole-arm translocation. The breakages were commonly situated in either terminal regions or centric regions. In comparison, a total of 104 translocation lines were identified in our study. The breakpoints were distributed along the entire length of chromosome 6**P**, resulting in various types of alien translocation including fragmental translocation, whole-arm translocation and intercalary translocation.

Efficient repair of double-strand breaks (DSBs) is critical for the survival and inheritance of all genomic DNA. DSBs could be repaired by nonhomologous end joining (NHEJ) or homologous recombination (HR) [27]. The mechanism of chromosome ends fusion can provide new insights into chromosomal rearrangement and genome evolution. Zhang et al [28] isolated an alloplasmic plant with “zebra” chromosome z5A in the *Elymus* *trachycaulus* */Triticum aestivum* backcross derivative. Chromosome z5A contained four 1H^t^ segments and five 5A segments in its formation that might have derived from nonhomologous recombination during the DNA DSB repair process. More than one DSB might occur in the genome when plants are exposed to irradiation. Those chromosome fragments could be stable through similar end sequences fusion. The chromosome (pointed by arrow) in Figure 2f contained two 6**P** chromosome segments (red color) and three wheat chromosome segments (blue color). It was similar to chromosome z5A and needed multiple fracture of wheat and 6**P** chromosome.

In the present study, the large number of various translocations obtained will facilitate our evaluation of the mechanism of alien chromosome translocation and the effect of gene dosage (repeat or deletion). In addition, they are valuable materials for investigating the relationship between breakpoints and genes in adjacent chromosome regions [29].

### Usefulness of various types of alien translocation in breeding

Common wheat is an important source of nutrient elements and accounts for 20% of the calories consumed by humans [30]. With the proliferation of the world population, global food requirements need to increase by 70–110% by 2050 [31]. The decreasing farmlands raise the need of per-hectare yield of cereal crop. It is imperative to improve the yield of the common wheat.

Previously, breeders raised wheat yield mainly through transfer of pest resistance genes rather than high-yield gene. Although 4HL.5DL translocation line exhibited supernumerary spikelet character, the number of seeds per plant was not increased [32]. As wheat- *Psathyrostachys* *huashanica* 6Ns disomic addition had the trait of twin spikelets [33], creating stable translocation line carring this trait might be used to improve wheat yield. Increased yields in the 1**RS**/1**BL** wheat cultivars may be due to a major gene or genes on l**RS**, or to a heterotic effect of the presence of rye chromatin [34,35]. *A* *. cristatum* chromosome 6**P** contains the gene(s) controlling the high numbers of florets and kernels per spike. Incorporating the gene(s) into wheat will conntribute to the improvement of wheat yield.

The wild relatives have a lot of superior agronomic traits and are valuable gene resources for wheat genetic improvement [36]. Despite some remarkable successes, alien gene introgression remains laborious, ineffective and largely unfulfilled [37]. There are many reasons for this situation. Deleterious alien genes are introduced along with the targeted desirable genes, namely linkage drag [24,38,39]. Noncompensating translocations cause duplications and deficiencies that are usually agronomically undesirable [35]. Wheat genetic balance is broken and alien genes cannot compensate for the loss of wheat chromosome segments.

Production of wheat- *A* *. cristatum* translocation lines is the best way to confer the multikernel gene to wheat. The translocation lines obtained in this study differed in breakpoint locations and alien segment lengths. The same chromosome 6**P** segment could be transferred to different wheat chromosomes/genomes. Different chromosome 6**P** segments could be also transferred to the same wheat chromosome/homologous group. These will be helpful for the understanding of recombination, interaction and genetic balance between chromosome 6**P** and wheat chromosomes. In addition, they can provide a scientific basis for the utilization of desirable genes of chromosome 6**P** in wheat breeding.

The efficient method of inducing wheat- *A* *. cristatum* translocation can offer a reference for the production of wheat-relative species translocation. Wheat- *A* *. cristatum* translocation lines are of significance to both basic research and breeding application.
